# Non-specific electrocardiographic ST-T abnormalities predict mortality in patients on peritoneal dialysis

**DOI:** 10.3389/fcvm.2022.930517

**Published:** 2022-12-15

**Authors:** Xiaojiang Zhan, Chuanfei Zeng, Jiajing He, Menghui Wang, Jun Xiao

**Affiliations:** ^1^Department of Nephrology, The First Affiliated Hospital of Nanchang University, Nanchang, Jiangxi, China; ^2^Jiangxi Medical College, Nanchang University, Nanchang, Jiangxi, China

**Keywords:** electrocardiogram, non-specific ST-T abnormalities, mortality, cardiovascular diseases, peritoneal dialysis

## Abstract

**Background:**

This study aimed to evaluate the predictive value of non-specific ST-segment and/or T-wave abnormalities in electrocardiography (ECG) for all-cause and cardiovascular mortality (CVM) in peritoneal dialysis (PD) patients.

**Methods:**

All patients who started PD between November 1, 2005, and February 28, 2017, at the First Affiliated Hospital of Nanchang University were enrolled. The primary outcomes were all-cause mortality and CVM. The Kaplan–Meier method and a log-rank test were used for the survival analysis. Multivariate Cox proportional hazards models were used to investigate the risk factors for all-cause mortality and CVM.

**Results:**

A total of 724 eligible PD patients were enrolled, including 401 (55.4%) men. In total, 153 (21.1%) patients died during a mean follow-up period of 27 (interquartile range, 13–41) months, and cardiovascular death was responsible for 84 of these deaths. The patients with non-specific ST-T abnormalities (NSSTTAs) had lower overall and cardiovascular survival rates compared to those free from any ECG abnormalities. According to the multivariate Cox proportional hazards models, (NSSTTAs) are independent risk factors for all-cause mortality and CVM, the hazard ratios are 1.81 (95% confidence interval, 1.11–2.95; *p* = 0.017) and 2.86 (95% confidence interval, 1.52–5.37; *p* = 0.001), respectively.

**Conclusion:**

Non-specific ST-T abnormalities can serve as risk markers of all-cause and CVM in PD patients.

## 1 Introduction

Chronic kidney disease (CKD) is a leading cause of death worldwide, with an estimated global incidence of 11–13% (1). Cardiovascular diseases (CVDs) are common complications in patients with CKD, and dialysis patients are at the greatest risk for CVDs including ventricular arrhythmias and sudden cardiac death (SCD). According to statistics, approximately 55.2% of patients on dialysis have CVDs, and 44.2% experience cardiac arrest or arrhythmias (2). In addition, CVDs are the leading cause of mortality in patients undergoing peritoneal dialysis (PD), accounting for > 40% of deaths in this population (3–5). Previous studies have shown that traditional factors like serum cholesterol, blood pressure, diabetes, and smoking are common risk factors for cardiovascular mortality (CVM) (6). However, despite the popularization of prevention measures, PD patients remain at increased risk for CVM. Therefore, the early identification of CVD risk groups may have prominent clinical significance for improving risk stratification and treatment management.

Electrocardiography (ECG) is a simple, non-invasive method that has been used to diagnose coronary heart disease (CHD) and CVD for nearly 100 years (7). Isolated non-specific ST-segment abnormalities, isolated non-specific T-wave abnormalities, and non-specific ST-T abnormalities (NSSTTAs) are the most common non-specific ECG abnormalities found in otherwise healthy people (8–10). NSSTTAs may be characterized by elevated or depressed ST-segments or changes in T-waves, such as their slight flattening, which can suggest subclinical CHD, early left ventricular hypertrophy, increased left ventricular weight, or an autonomic nervous imbalance (11). NSSTTAs account for most of the ST-T abnormalities revealed via ECG investigation. Slight ST-T abnormalities are observed in approximately 1% of resting ECGs in the healthy population and may be associated with an increased risk of death (12). Cho et al. (13) investigated 16,793 Korean active subjects, including 1,037 subjects with NSSTTAs who had a greater incidence of atrial fibrillation or flutter at the 10-year follow-up point compared to those without NSSTTAs. In a prospective study, Greenland et al. (14) followed 7,985 women and 9,630 men for 22 years and found that a single mild ST-segment depression, mild T-wave abnormality, or mild ST-T abnormality was associated with an increased risk of death and had long-term prognostic effects on CVM. Such abnormalities could be used as markers of an increased risk of CHD and CVD. Nevertheless, studies of the predictive value of non-specific ST-segment and/or T-wave abnormalities on ECGs in dialysis patients are limited. The purpose of this study was therefore to assess the relationship between non-specific ST-segment and/or T-wave abnormalities and all-cause mortality and CVM in PD patients.

## 2 Materials and methods

### 2.1 Study design and baseline data collection

Between November 1, 2005, and February 28, 2017, we selected dialysis patients receiving PD at the First Affiliated Hospital of Nanchang University in Jiangxi province, China. Patients aged > 18 years, and PD was maintained for ≥ 3 months after the first PD session were enrolled, while those with conversion from hemodialysis, failed kidney transplantation, lack of ECG data, and premorbid CVD were excluded. We also declined to enroll patients with abnormal ECGs and secondary ST-T changes, such as probable myocardial infarction, atrial fibrillation, atrioventricular block, or premature contraction. All data and study participants were approved by the Human Ethics Committee of Nanchang University [Application ID: (2021) 9-021], and this study conformed with the ethical principles of the Declaration of Helsinki (15).

Participants were divided into 4 groups according to the ECG reports, as follows: patients with normal ECGs, those with isolated non-specific ST-segment abnormalities, those with isolated non-specific T-wave abnormalities, and those with NSSTTAs (ECG reports are provided in Supplementary Figures 1–4). All patients were followed up until the cessation of PD, death, or May 31, 2017. Baseline data and biochemical indicators, including age, sex, body mass index (BMI), diabetes, hypertension, CVD, hemoglobin, albumin, total cholesterol (TC), triglycerides (TGs), low-density lipoprotein cholesterol (LDL-C), and high-density lipoprotein cholesterol (HDL-C), were collected from all participants within the first 3 months of PD. Data collected before the catheterization day were preferred. The baseline residual renal function was evaluated using the glomerular filtration rate (eGFR) estimated by CKD epidemiology combined with the creatinine equation before commencing PD (16). According to the American Heart Association, the causes of CVM include acute myocardial infarction, SCD, heart failure, stroke, and cardiovascular bleeding (17).

### 2.2 Definition of non-specific ST-segment and/or T-wave abnormalities

Standard 12-lead ECGs were collected before PD catheter insertion or within 3 months of PD. The records were evaluated by an ECG specialist and reviewed by a supervising doctor. Non-specific ST-segment and/or T-wave abnormalities on ECGs were defined according to Minnesota Codes (MC). Criteria are as follows: isolated non-specific ST-segment abnormalities (MC 4-3 or 4-4); isolated non-specific T-wave abnormalities (MC 5-3 or 5-4); non-specific ST-T abnormalities (MC 4-3 or 4-4 plus MC 5-3 or 5-4) (18).

### 2.3 Statistical analyses

SPSS software version 26 (IBM Corporation, Armonk, NY, USA) and R version 4.0.2 (R Foundation for Statistical Computing, Vienna, Austria)^1^ were used to calculate all variable data. Categorical variables are presented as frequencies and percentages, normally distributed continuous variables are presented as mean ± standard deviation values, and non-normally distributed continuous variables are presented as medians and interquartile ranges. One-way analysis of variance, the Kruskal–Wallis test, Kaplan–Meier analysis, and multivariate Cox proportional hazards models were used for statistical analysis. This study fit a total of 3 multivariate proportional hazards models. Covariates with *p* < 0.05 in the univariate Cox analysis or that were deemed to be clinically significant were chosen for multivariate Cox proportional hazards regression. Age, sex, BMI, premorbid diabetes, and hypertension were adjusted in model 1. Hemoglobin, albumin, uric acid, TC, HDL-C, LDL-C, and potassium were further adjusted in model 2. Finally, anti-hypertensive medication use was adjusted in model 3. The results are presented as hazard ratio (HR) and 95% confidence interval (95% CI) values, and *p* < 0.05 indicates statistical significance.

## 3 Results

### 3.1 Baseline patient characteristics

This study eventually included 724 incident PD patients. In total, 351 patients had normal ECG findings, 174 patients had isolated non-specific T-wave abnormalities, 39 patients had isolated non-specific ST-segment abnormalities, and 160 patients had NSSTTAs. Comparisons of baseline data and the biochemical indicators of all participants are shown in Table 1. This study revealed no significant differences among the groups with different ECG abnormalities in terms of age, sex, BMI, premorbid diabetes, hypertension, eGFR, total Kt/V, albumin, or lipids, with hemoglobin levels being an exception (Table 1).

**TABLE 1:** Baseline characteristics of participants with or without non-specific ST-segment and/or T-wave abnormalities.

Variables	Normal ECG finding (*n* = 351)	Non-specific T-wave abnormalities (*n* = 174)	Non-specific ST-segment abnormalities (*n* = 39)	Non-specific ST-T abnormalities (*n* = 160)	*P*-value
Age (years)	48.4 ± 14.2	46.7 ± 13.8	51.8 ± 12.3	47.5 ± 13.9	0.175
Male sex (%)	188 (53.6)	93 (53.4)	21 (53.8)	99 (61.9)	0.321
Body mass index (kg/m^2^)	21.7 ± 3.4	22.1 ± 3.7	21.3 ± 2.8	21.9 ± 3.4	0.561
Diabetes (%)	50 (14.2)	29 (16.7)	7 (17.9)	35 (21.9)	0.200
Hypertension (%)	246 (70.1)	117 (67.2)	28 (71.8)	126 (78.8)	0.111
eGFR (mL/min/1.73 m^2^)	3.2 (1.7–5.5)	2.8 (1.7–4.9)	3.1 (2.2–4.3)	3.1 (1.6–6.0)	0.531
Total Kt/V	2.2 (1.7–2.7)	2.3 (1.7–2.7)	2.1 (1.4–2.6)	2.1 (1.7–2.7)	0.782
Hemoglobin (g/dL)	77.1 ± 15.6	78.3 ± 16.6	71.5 ± 16.5	81.7 ± 18.6	0.002
Albumin (g/L)	35.5 ± 5.5	35.6 ± 5.5	36.7 ± 3.9	35.5 ± 5.6	0.642
Total cholesterol (mmol/L)	4.16 ± 1.30	4.21 ± 1.17	4.14 ± 1.25	4.34 ± 1.10	0.499
Triglyceride (mmol/L)	1.27 (0.89–1.79)	1.25 (0.88–1.80)	1.37 (0.99–1.81)	1.37 (0.89–1.87)	0.742
HDL-C (mmol/L)	1.09 (0.91–1.40)	1.09 (0.92–1.43)	1.07 (0.78–1.15)	1.19 (0.91–1.46)	0.110
LDL-C (mmol/L)	2.22 (1.78–2.85)	2.41 (1.91–2.98)	2.23 (1.66–2.87)	2.41 (1.89–2.93)	0.121
Potassium (mmol/L)	4.4 ± 0.8	4.5 ± 0.8	4.2 ± 0.9	4.4 ± 0.8	0.071

HDL-C, high-density lipoprotein cholesterol; LDL-C, low-density lipoprotein cholesterol. In all cases, *p* < 0.05 is considered statistically significant.

### 3.2 Prognostic value of baseline non-specific ST-segment and/or T-wave abnormalities

**FIGURE 1:**
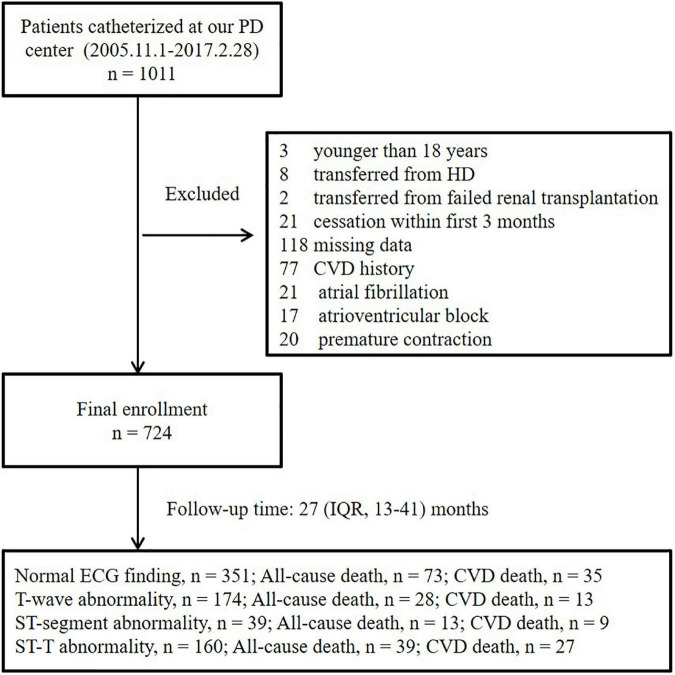
Enrollment flowchart. PD, peritoneal dialysis; HD, hemodialysis; IQR, interquartile range; ECG, electrocardiogram; CVD, cardiovascular disease.

After a median follow-up period of 27 (interquartile range, 13–41) months, 73 all-cause deaths, including 35 CVD deaths, were recorded among the patients with normal ECG findings; 28 all-cause deaths, including 13 CVD deaths, were recorded among the patients with isolated non-specific T-wave abnormalities; 13 all-cause deaths, including 9 CVD deaths, were recorded among the patients with isolated non-specific ST-segment abnormalities; and 39 deaths, including 27 CVD deaths, were recorded among the patients with NSSTTAs, respectively (Figure 1).

**FIGURE 2:**
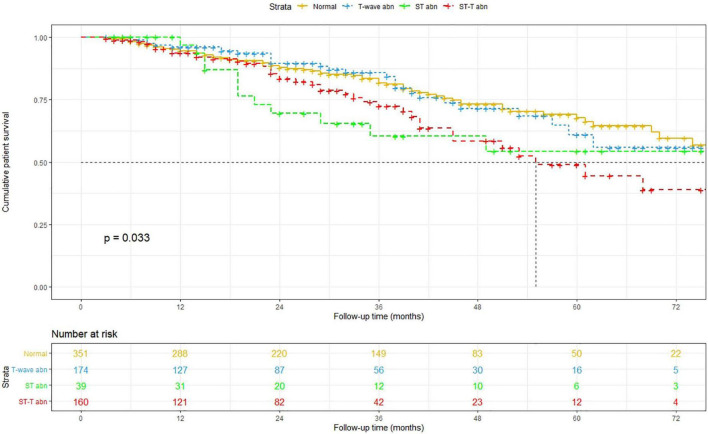
Kaplan–Meier curves for the cumulative patient survival.

**FIGURE 3:**
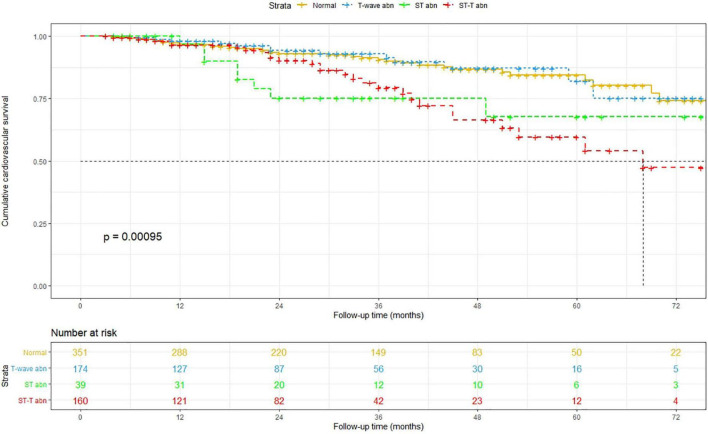
Kaplan–Meier curves for the cumulative cardiovascular survival.

The Kaplan–Meier method was used to estimate overall and cardiovascular survival rates of the patients with NSSTTAs and those free from any ECG changes (Figures 2, 3). The participants who presented with NSSTTAs had significantly lower cumulative overall and cardiovascular survival rates compared to those with normal ECG findings (log-rank *p* < 0.05).

The long-term prognostic effect of non-specific ST-segment and/or T-wave abnormalities on all-cause mortality and CVM was assessed using Cox proportional hazards models (Table 2). Compared to the PD patients with normal ECG findings, those with NSSTTAs had HRs (95% CIs) for all-cause mortality and CVM of 1.70 (1.06–2.72) and 2.87 (1.55–5.30) after adjusting for age, sex, BMI, premorbid diabetes, and hypertension in model 1; 1.80 (1.10–2.92) and 2.87 (1.53–5.37) after further adjusting for hemoglobin, albumin, uric acid, triglyceride, HDL-C, LDL-C, and potassium in model 2; and 1.81 (1.11–2.95) and 2.86 (1.52–5.37) after further adjusting for anti-hypertensive medication use in model 3, respectively (Table 2).

**TABLE 2:** The associations of ST-segment and/or T-wave abnormalities with all-cause and CVM.

Variables	T-wave abnormalities		ST-segment abnormalities		ST-T abnormalities	
	**HR (95% CI)**	***P*** **-value**	**HR (95% CI)**	***P*** **-value**	**HR (95% CI)**	***P*** **-value**
**All-cause mortality**						
Model 1^a^	1.08 (0.61–1.93)	0.797	2.00 (0.98–4.09)	0.058	1.70 (1.06–2.72)	0.027
Model 2^b^	1.02 (0.57–1.84)	0.943	1.97 (0.94–4.12)	0.073	1.80 (1.10–2.92)	0.018
Model 3^c^	1.05 (0.58–1.88)	0.881	1.83 (0.84–3.98)	0.126	1.81 (1.11–2.95)	0.017
**Cardiovascular mortality**						
Model 1^a^	1.13 (0.49–2.58)	0.778	3.01 (1.21–7.51)	0.018	2.87 (1.55–5.30)	0.001
Model 2^b^	1.04 (0.45–2.40)	0.937	3.02 (1.18–7.74)	0.022	2.87 (1.53–5.37)	0.001
Model 3^c^	1.04 (0.45–2.42)	0.930	3.25 (1.26–8.39)	0.015	2.86 (1.52–5.37)	0.001

HR, hazard ratio; CI, confidence interval; BMI, body mass index; CVM, cardiovascular mortality.

^a^Model 1: adjusted for age, sex, BMI, premorbid diabetes, and hypertension. ^b^Model 2: model 1 adjusted also for hemoglobin, albumin, uric acid, triglycerides, high-density lipoprotein cholesterol, low-density lipoprotein cholesterol, and potassium. ^c^Model 3: model 2 adjusted also for anti-hypertensive medication use.

Isolated non-specific ST-segment abnormalities were associated with CVM even after full adjustment in model 3, and the HR (95% CI) was 3.25 (1.26–8.39). However, isolated non-specific ST-segment abnormalities were not associated with all-cause mortality; moreover, isolated non-specific T-wave abnormalities were not associated with either all-cause mortality or CVM in models 1–3 (Table 2).

## 4 Discussion

This study is the first to show that NSSTTAs are a new predictive risk factor for all-cause mortality and CVM in PD patients. We analyzed 4 groups of patients with normal ECGs, isolated non-specific T-wave abnormalities, isolated non-specific ST-segment abnormalities, and NSSTTAs, respectively, and found that no significant differences existed in age, sex, BMI, premorbid diabetes, hypertension, eGFR, total Kt/V, albumin, or lipids among the groups, with hemoglobin level being an exception. In addition, the PD patients with NSSTTAs had significantly lower cumulative overall and cardiovascular survival rates. Therefore, we suggest that NSSTTAs may provide a new strategy for the non-invasive prediction of all-cause mortality and CVM in patients receiving PD.

NSSTTAs are common in healthy people, and NSSTTAs in individuals with non-renal diseases have shown a good ability to predict cardiovascular morbidity and mortality. Sawano et al. (19) investigated 14,077 participants, including 3,111 (22.1%) that had NSSTTAs at baseline. Their results showed that 106 patients (3.4%) with NSSTTAs had experienced ischemic stroke, and NSSTTAs were associated with a 32% increased risk of ischemic stroke after model adjustment. Badheka et al. (20) revealed a positive relationship between NSSTTAs and higher incidence rates of CVM and all-cause mortality in a large nationally representative cross-sectional cohort study. Furthermore, the association between NSSTTAs and CVD or CHD is not affected by traditional risk factors (21). The underlying mechanisms explaining the association between non-specific NSSTTAs and cardiovascular morbidity and mortality have not been clarified. Some studies have suggested that NSSTTAs may indicate the presence of subclinical CHD, early left ventricular hypertrophy, an increased left ventricular weight, or an autonomic nervous imbalance (11, 21). However, other studies did not separate isolated ST-segment abnormalities and isolated T-wave changes from NSSTTAs. Moreover, patients with renal failure are prone to cardiovascular changes, but few studies have focused on the role of non-specific ECG changes in this population.

Dialysis patients usually experience changes in hemodynamics, electrolytes, and the acid–base balance, all of which are potentially arrhythmogenic. Abe et al. (22) found that, among the 142 Holter records of 72 hemodialysis patients, ST-T abnormalities were found in 43 cases (60%). ST-segment depression occurred in 11 patients during and several hours after dialysis, and dynamic ECGs showed a high incidence of arrhythmias and ST-T abnormalities. Kalcik et al. (23) previously reported that the duration of the P-wave, QRS complex, and R-wave peak time were significantly increased in patients with end-stage renal disease. Also, Kuo et al. (24) found that 196 (64%) of 306 PD patients had prolonged QT intervals. The clinical significance of non-specific ECG changes in dialysis patients is consistent with that in the general population. Meanwhile, Omae et al. (25) determined that ST-T changes predict the cardiovascular outcomes of chronic hemodialysis patients, and Jaroszynski et al. (26) concluded that a positive T-wave in lead aVR is an independent and powerful predictor of CVM in hemodialysis patients. In patients with end-stage renal disease, other investigators have found that QT interval, spatial QRS-T angle, signal averaged ECG, heart rate variability, and T-wave alternation may be related to the cardiac risk (27, 28).

In this study, we analyzed the relationship between different non-specific ECG changes and CVM and all-cause mortality, and the results showed that patients who presented with NSSTTAs tended to have higher hemoglobin levels. This seems to be contrary to the results of a previous study, which reported that lower hemoglobin levels are an independent risk factor for CVD and mortality in patients with CKD (29). We suppose that this result may be related to the greater proportion of men with NSSTTAs, as previous studies have reported that male dialysis patients typically have higher hemoglobin levels than their female counterparts (30). In addition, we also found that isolated ST-segment abnormalities and NSSTTAs predict CVM and all-cause mortality in PD patients, but we failed to find such a relationship between isolated T-wave abnormalities and CVM and all-cause mortality. To the best of our knowledge, this is a novel finding. Although the underlying pathophysiology mechanism remains unknown, we suspect that non-specific ST-segment or ST-T abnormalities may reflect subclinical cardiovascular changes, as many factors—such as anemia, a micro-inflammatory state, uremic toxin accumulation, fluid overload, secondary hyperparathyroidism, hypertension, altered lipid metabolism, and the accumulation of gut microbiota–derived uremic toxins like trimethylamine N-oxidase—may affect cardiovascular function in the context of renal failure (31). Nevertheless, T-waves are more susceptible to electrolyte disturbances, especially changes in potassium, which are common in patients undergoing PD (29, 32) and may eventually mitigate the T-wave’s effect on the prognosis of PD patients.

There are some limitations of our research. First, our study was a single-center retrospective study that enrolled only a small number of PD patients. Second, the appearance of NSSTTAs can be linked to transient physiological phenomena, abnormal left ventricular wall motion without CHD, electrolyte disorders, and drug use (33), limiting the repeatability of identifying NSSTTAs on continuous ECG measurements. Third, the level of inflammatory factors will also affect changes in ECGs and influence the all-cause mortality and CVM of PD patients. Therefore, we will further explore the role and risk factors of NSSTTAs in PD patients and whether inflammatory factors are involved in NSSTTAs in the future.

## 5 Conclusion

In summary, non-specific ECG changes are common in PD patients. Our results found that NSSTTAs are associated with long-term all-cause mortality and CVM in patients with PD.

## Data availability statement

The raw data supporting the conclusions of this article will be made available by the authors, without undue reservation.

## Ethics statement

The studies involving human participants were reviewed and approved by the Human Ethics Committee of Nanchang University. Written informed consent for participation was not required for this study in accordance with the national legislation and the institutional requirements.

## Author contributions

XZ conceived and designed the study and performed the statistical analyses. JH and MW collected the data. CZ drafted the manuscript. JX made revisions to the manuscript. All authors read and approved the final manuscript.

## Footnotes

1
http://www.r-project.org
