# Supplementary figures and images for: Non-specific electrocardiographic ST-T abnormalities predict mortality in patients on peritoneal dialysis
Source: Front Cardiovasc Med. 2022 Dec 15;9:930517. doi: 10.3389/fcvm.2022.930517 (PMC9798218; doi:10.3389/fcvm.2022.930517)

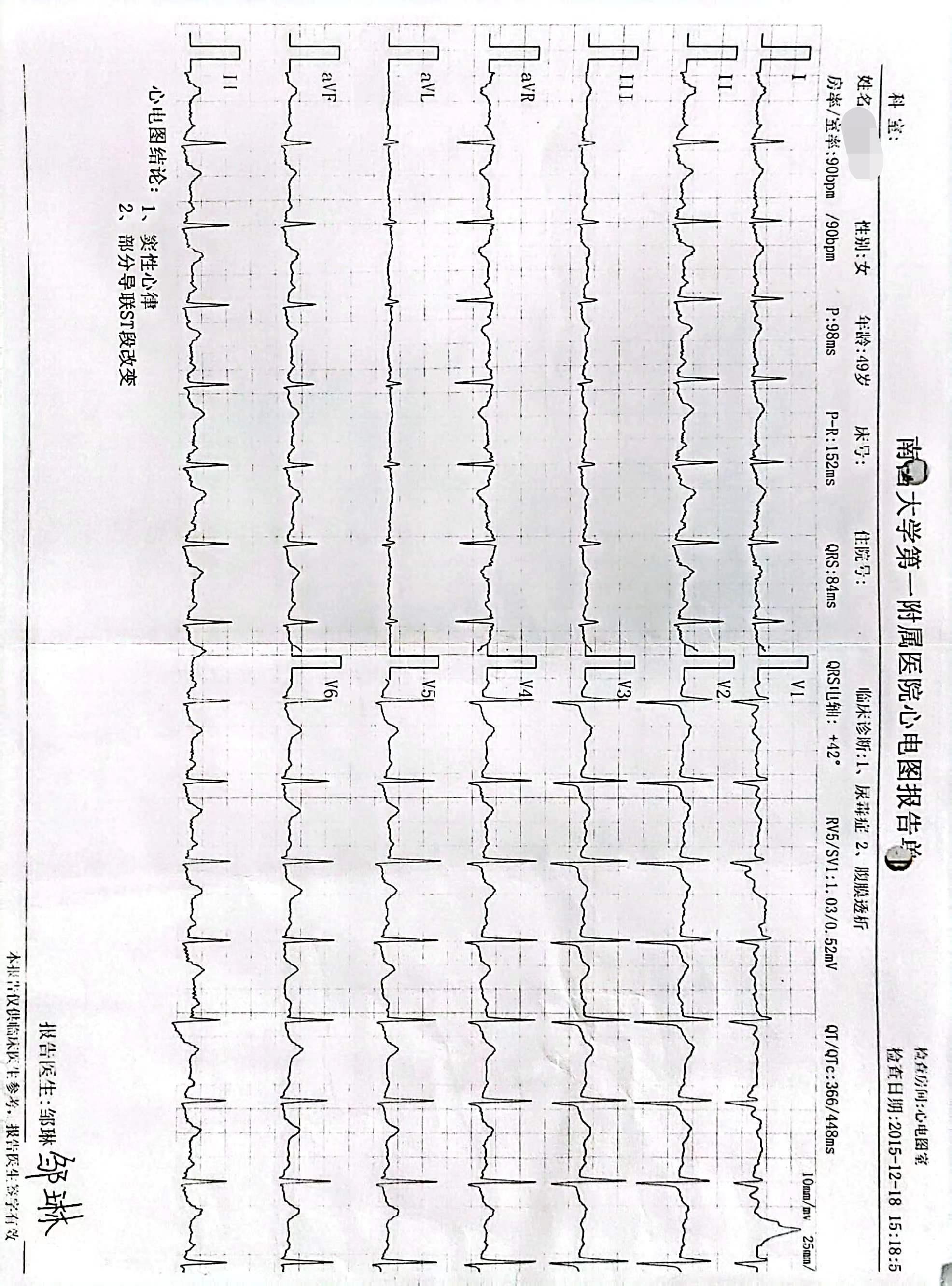

Supplement: Supplementary file 1 [file Image_1.JPEG]

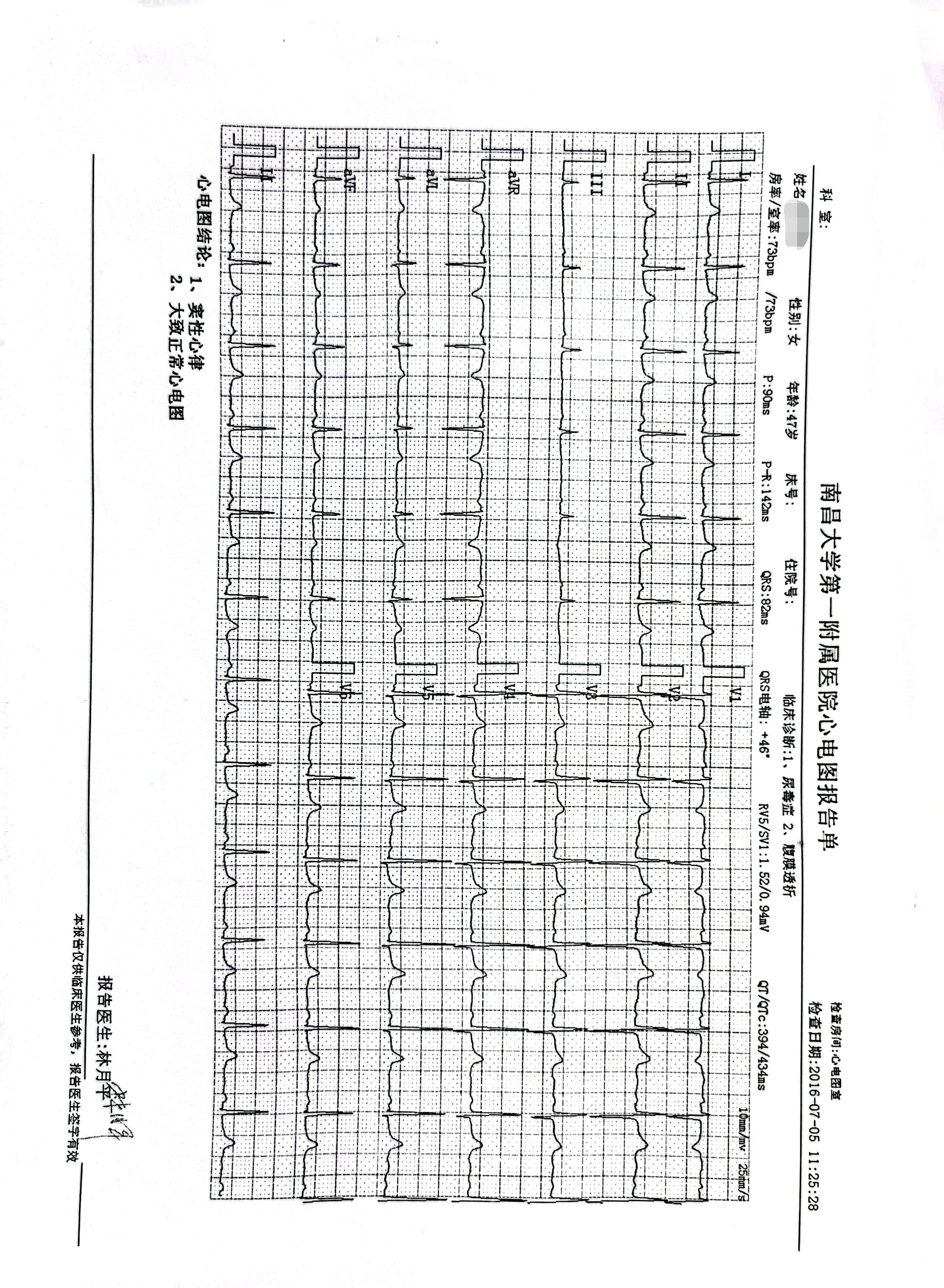

Supplement: Supplementary file 2 [file Image_2.JPEG]

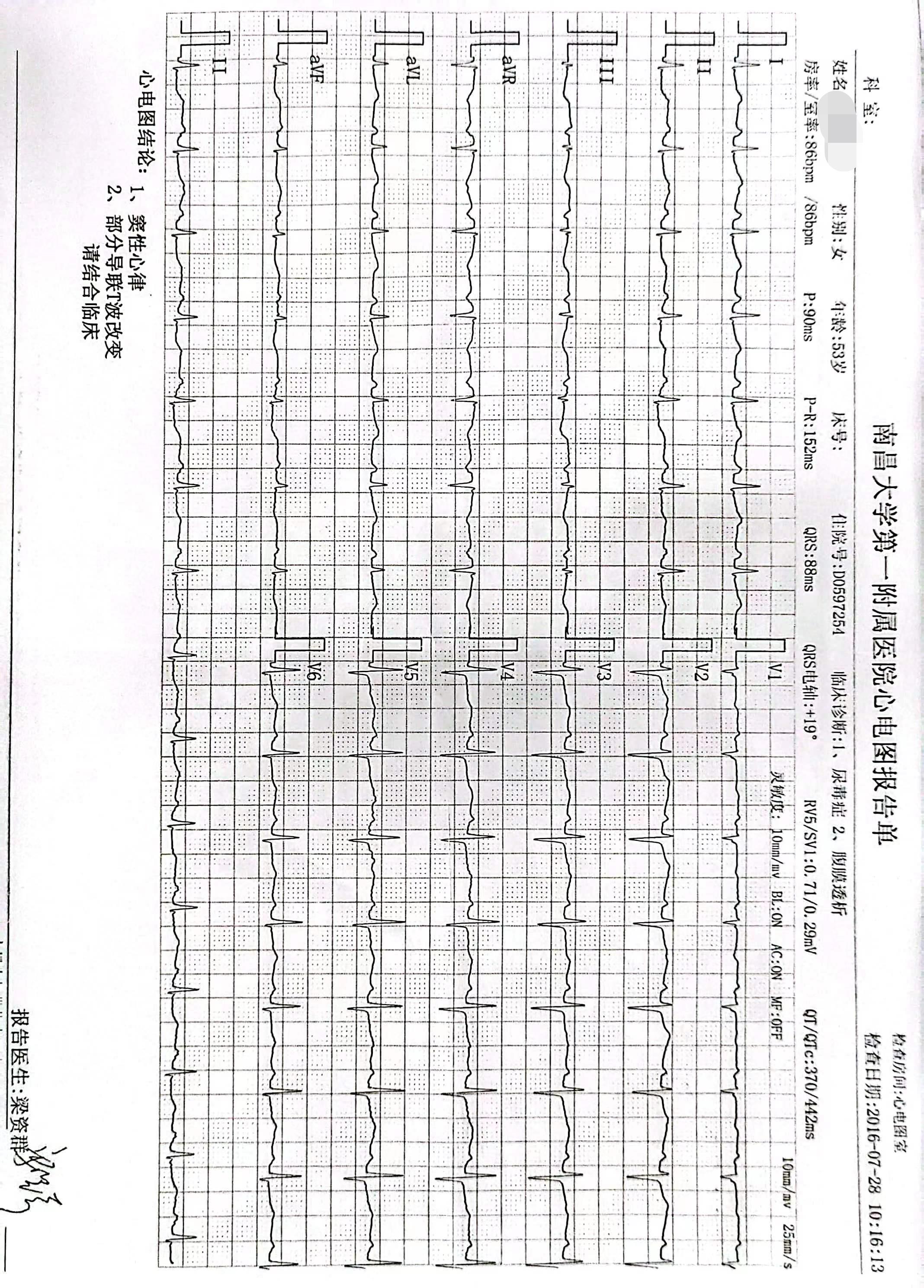

Supplement: Supplementary file 3 [file Image_3.JPEG]

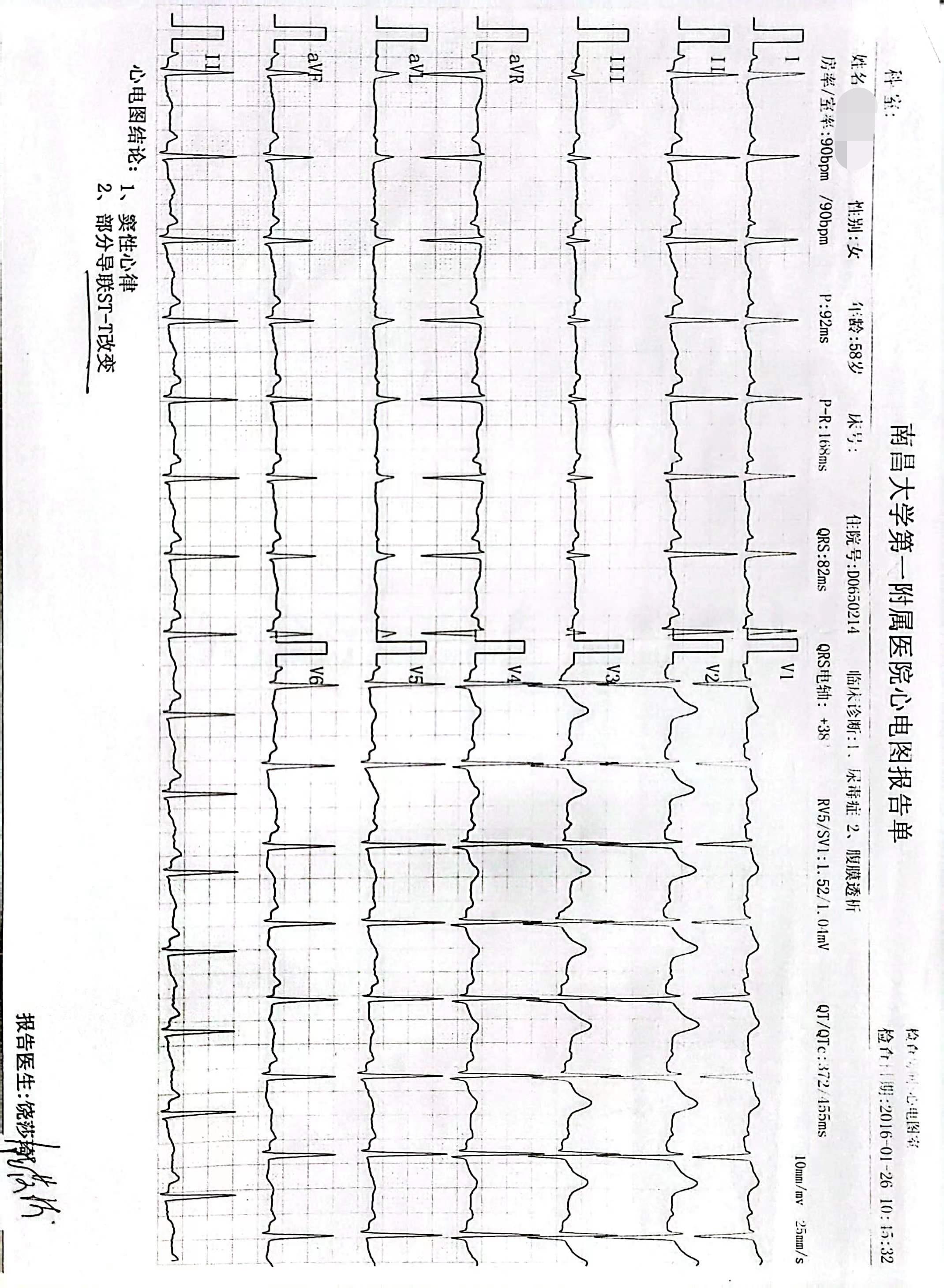

Supplement: Supplementary file 4 [file Image_4.JPEG]
